# Phase Segmentation and Phase-Specific Kinematic Feature Extraction of Hurdle Clearance Based on Monocular Video and Markerless Pose Estimation

**DOI:** 10.3390/s26123822

**Published:** 2026-06-16

**Authors:** Yuxin Guo, Shaoze Zheng, Chen Liu, Huashuai Li

**Affiliations:** College of Physical Education and Sports, Beijing Normal University, Beijing 100875, China; 202231070020@mail.bnu.edu.cn (Y.G.); 202522070173@mail.bnu.edu.cn (C.L.)

**Keywords:** hurdling, monocular video, markerless pose estimation, phase segmentation, phase-specific kinematic features, biomechanics

## Abstract

Hurdle technique analysis requires accurate identification of key phases and kinematic features, but conventional biomechanical methods are often costly, equipment-dependent, and difficult to apply in front-line training. This study developed a low-cost monocular-video-based framework for rapid hurdle clearance analysis in practical training settings. Thirty-seven physical education college students with different hurdling skill levels were recruited as participants, and side-view videos of their hurdle clearance were recorded. The proposed pipeline combined YOLO26 hurdle detection, RTMPose markerless pose estimation, rule-based key-event detection, phase segmentation, and phase-specific kinematic feature extraction. The results showed that the hurdle detection model achieved high accuracy, with bounding-box mAP@0.5 of 0.992 and mask mAP@0.5 of 0.971. Pose estimation showed good agreement with manual annotations, with an overall RMSE of 8.25 px and PCK of 97.64%. The rule-based phase segmentation method achieved an overall event localization MAE of 0.74 frames and RMSE of 1.55 frames, outperforming LSTM and TCN temporal baselines. Core distance and most angle variables also showed high agreement with manually recalculated values. These findings indicate that monocular video and markerless pose estimation can provide an accurate, low-cost, and practical tool for hurdle phase segmentation and kinematic assessment in routine training contexts.

## 1. Introduction

Hurdling combines sprint speed, rhythm control, and complex event-specific technique and places high demands on speed ability and movement coordination. Its technical essence is to minimize horizontal velocity loss during hurdle clearance, shorten flight time, optimize the trajectory of the body center of mass, and maintain a smooth transition between the approach and post-hurdle running rhythm [1]. Previous studies have established a relatively clear set of core kinematic indicators for hurdle analysis, mainly including spatiotemporal and joint kinematic variables within the hurdle unit, such as takeoff distance; landing distance; hurdle step length; flight time; support time; takeoff angle; trunk inclination; hip, knee, and ankle joint angles; and center-of-mass displacement and velocity before takeoff and during hurdle clearance [1,2,3,4].

Although the main indicators used to evaluate hurdling technique are relatively clear, current methods commonly rely on multiple high-speed cameras combined with commercial software, such as Dartfish or SIMI Motion, for frame-by-frame manual digitization or on direct linear transformation (DLT) procedures to convert two-dimensional coordinates into three-dimensional data for kinematic analysis [5,6,7]. These approaches can provide accurate measurements, but they require specialized expertise, complex procedures, and substantial manual effort, which limits their use in routine training environments.

Electromyography, inertial sensors, and infrared motion capture systems are now well developed in sports biomechanics, but they have rarely been combined with hurdling technique analysis [8,9,10,11,12]. This may be because the primary goal of hurdling research is often to establish performance-related variables rather than to reconstruct complete neuromuscular control mechanisms [4]. In addition, hurdling studies rely heavily on competition or near-competition ecological settings, where field data collection constrains the deployment of biomechanical equipment [13,14]. Because participants in hurdling studies are usually athletes, research designs also tend to prioritize kinematic observation methods that minimally interfere with competitive state and training performance [15].

With the rapid development of artificial intelligence, markerless pose estimation has become increasingly mature. Jafarzadeh et al. (2021) first applied OpenPose directly to pose estimation in hurdle athletes, requiring only video recordings without attaching markers or sensors to the body and showing potential for near-real-time feedback [16]. Their later study comparing YOLOv8 (Ultralytics Inc., Los Angeles, CA, USA) and DeepLabCut (version 2.2.2, Mathis Laboratory, École Polytechnique Fédérale de Lausanne, Lausanne, Switzerland) in hurdle training videos further suggested that markerless pose estimation can support coaches and that effective analytical tools are emerging, although stable and generalizable software tools remain limited [17]. Needham et al. (2021) also noted that marker-based motion capture, although regarded as a gold standard, is restricted to laboratory conditions and is difficult to apply widely; they recommended caution when using markerless methods in specialized movement tasks and suggested event-specific retraining [18]. Chen et al. (2025) reported that monocular video methods still face challenges such as occlusion, motion blur, lighting variation, and real-time computational load, but recent developments have enabled deployment on mid-range GPUs and CPUs, indicating that markerless motion capture is becoming increasingly practical [19].

Taken together, the key indicators for hurdling technique analysis are well established, but existing acquisition methods often depend on specialized equipment, complex field setup, or extensive frame-by-frame manual processing, making them difficult to use for low-cost and rapid feedback in training. At the same time, advances in markerless motion capture provide a new pathway for analyzing hurdling technique from ordinary videos. Therefore, this study addresses whether a low-cost software system suitable for training settings can automatically extract key technical variables from smartphone or camera videos of hurdle clearance and provide a practical tool for hurdling technique analysis. The proposed framework aims to reduce equipment requirements and manual workload while improving the feasibility of routine field-based analysis.

## 2. Materials and Methods

### 2.1. Study Design

**Figure 1:**
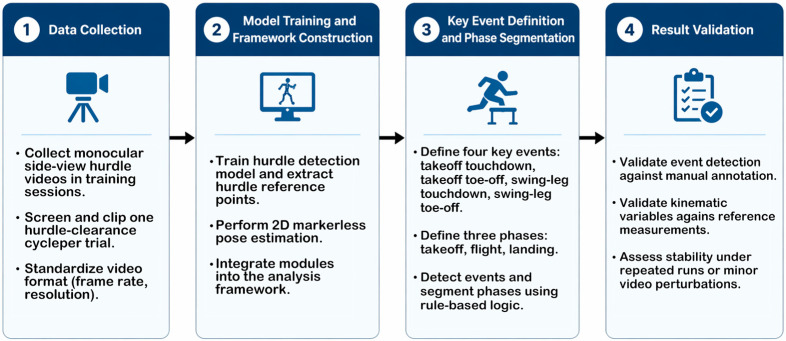
Overall workflow of the proposed monocular video analysis pipeline.

This methodological study developed a low-cost software framework for rapid technical analysis in athlete training settings. The framework combines a YOLO26 [20] object detection and segmentation model with an RTMPose markerless pose estimation model and proposes an analysis method for extracting hurdling technique variables from two-dimensional monocular side-view videos. The workflow includes hurdle detection, two-dimensional human pose estimation, key-event-based phase segmentation, phase-specific kinematic feature extraction, and validation experiments. The experimental workflow is shown in Figure 1.

### 2.2. Participants and Video Samples

A total of 37 first- to fourth-year track-and-field students from a physical education college were recruited (Table 1). All participants had received systematic hurdling instruction, but their sex and performance level varied. This heterogeneity was intentionally included to capture common technical characteristics across athletes and to identify general rules for phase boundary detection.

**Figure 2:**
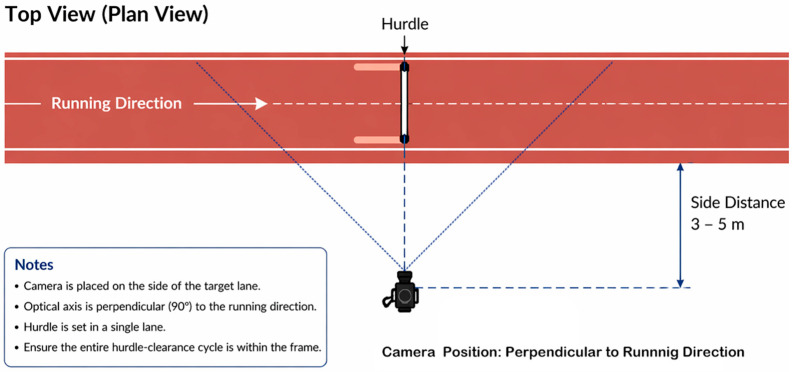
Representative side-view video sample for hurdle clearance analysis.

Participants were asked to sprint maximally and clear two consecutive hurdles. A pure side-view two-dimensional video of the second hurdle clearance was collected (Figure 2). Each video contained a complete hurdle-clearance cycle for subsequent key-event detection, phase segmentation, and phase-specific kinematic feature extraction. Because the main purpose of this study was method development and validation rather than between-participant comparison, participants were not further grouped by sex, competitive level, or training background; each video was treated as an independent analysis sample.

All videos were recorded from a monocular side-view perspective using a smartphone or camera. The frame rate was approximately 60 frames per second, and a relatively high shutter speed was used whenever possible to keep the athlete’s key body parts and hurdle structure clear within the same motion plane. The videos were required to cover the key process of hurdle clearance, including takeoff, flight, and landing phases, with the lower limbs and hurdle region visible throughout the main movement period to support frame-by-frame event detection and kinematic feature calculation.

To ensure analyzability and comparability, videos were included if they met the following criteria: (1) the camera view was approximately side-on to the hurdling action; (2) the video fully contained the takeoff, flight, and landing phases; (3) the athlete’s whole body and the hurdle structure were clearly visible and could support hurdle detection and two-dimensional pose estimation; and (4) the image quality was sufficient for frame-by-frame key-event judgment. Videos with severe occlusion, incomplete movement, or obvious motion blur that prevented reliable event detection were excluded. After screening, videos were preprocessed using stabilization, horizontal correction, and resolution normalization. A total of 37 videos were collected and used for hurdle detection, markerless pose estimation, key-event-driven phase segmentation, phase-specific feature extraction, and validation analyses.

### 2.3. Hurdle Detection

Unlike many other track-and-field events, automatic quantitative analysis of hurdle clearance requires evaluating the relative position between the athlete and the hurdle. Therefore, stable extraction of hurdle spatial information from video is necessary to build the reference coordinate information required for subsequent kinematic analysis.

YOLO26 was used as the front-end detection and segmentation model to identify the hurdle in the video and provide spatial constraints for subsequent markerless pose estimation and kinematic variable calculation. In each side-view monocular video, the system first automatically detected the hurdle, output the frame-wise bounding box, and further extracted the bar midpoint and post-reference position for event detection, phase segmentation, and spatial variable calculation.

**Figure 3:**
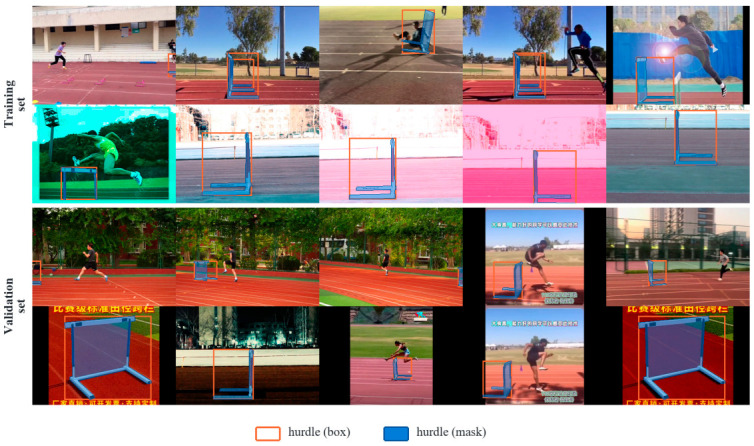
Hurdle annotation examples and model training dataset.

Model training was conducted using a self-built hurdle action dataset (Figure 3). Images were collected from self-recorded photographs, online images, and frames extracted from training videos, and the hurdle was manually annotated using the CVAT tool. The dataset was divided into training and validation sets, with 2187 training images and 438 validation images, totaling 2625 images; the training and validation proportions were 83.31% and 16.69%, respectively. Label statistics showed that 2149 training images contained valid annotations and 38 were unlabeled background frames, while 356 validation images contained valid annotations and 82 were unlabeled background frames. The dataset contained one category, named hurdle. Instance segmentation contour labels were used, allowing the training process to generate both object masks and bounding boxes to support object localization and region segmentation. To further evaluate the generalization ability of the model, frames were extracted at 30 fps from independently recorded hurdle-clearance videos of 37 athletes. A total of 1147 frames were obtained, and the hurdle regions were manually annotated to construct an independent test set. The final trained hurdle segmentation model was then evaluated on this test set.

A two-stage training strategy was adopted. In the first stage, the initial annotated dataset was used for baseline training so that the model could learn preliminary representations of the hurdle structure, human region, and hurdle-clearance posture. The dataset was then optimized based on the first-stage results, including correction of annotation errors, adjustment of boundary consistency, and addition of difficult samples. The first-stage best weights were then used to initialize the second-stage training, which was continued for 40 epochs. This strategy was intended to retain previously learned feature representations while further improving model stability and generalization under fast limb movement, hurdle occlusion, and complex clearance postures.

During training, validation-set performance was used for model selection. Bounding-box and segmentation metrics, including precision, recall, mAP@0.5, and mAP@0.5:0.95, were monitored, and box loss and segmentation loss were recorded to evaluate convergence. The retained training curves indicated that continued training after dataset optimization improved model performance.

After YOLO26 training, the model outputs were used for further analysis of hurdle-clearance videos. The frame-wise hurdle position, athlete region, and key movement phases provided visual priors for locating takeoff, flight, and landing events. Hurdle-relative spatial variables also provided additional information such as hurdle height. Thus, YOLO26 served as a core visual front-end in this study, improving the robustness and repeatability of the markerless motion analysis workflow through stable object detection and segmentation.

### 2.4. Human Pose Estimation

**Figure 4:**
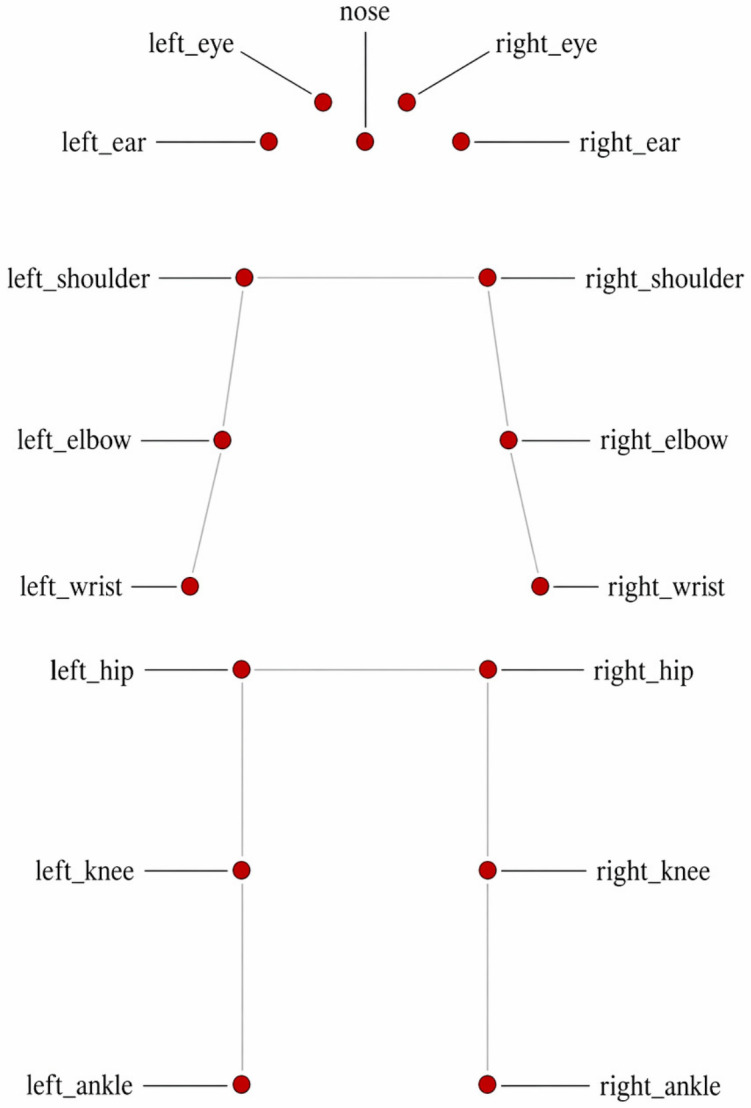
Body17 keypoint scheme used for human pose estimation.

The human pose estimation module was implemented using the MMPose inference framework. RTMDet-L was used as the detector, and an RTMPose two-dimensional pose model was used as the pose estimator. For the present analysis task, the system used the body17 keypoint scheme and output 17 body keypoints (Figure 4). The keypoints most directly related to subsequent hurdling analysis included the bilateral shoulders, hips, knees, and ankles. These keypoints were used for pelvis estimation, lower-limb joint angle calculation, trunk posture representation, and key-event detection.

To obtain time-series human movement information during hurdle clearance, two-dimensional markerless pose estimation was performed on the preprocessed videos. The side-view monocular video was used as input. A top-down pose estimation pipeline first detected the athlete’s body region and then estimated keypoints within the corresponding bounding box, outputting frame-wise two-dimensional keypoint coordinates and confidence scores.

Because multiple people or background interference may appear in hurdle videos, when more than one person was detected in a frame, the person with the largest bounding-box area was selected as the target athlete. This ensured that the keypoint sequence corresponded to the athlete performing the hurdle clearance. For frames without a valid person detection, empty keypoint placeholders were output to maintain consistent temporal length and support subsequent time-series alignment.

Pose estimation results were exported as frame-wise structured data, including frame number, timestamp, video frame rate, image size, person bounding-box coordinates, two-dimensional keypoint coordinates, and keypoint confidence scores. The pose results and hurdle detection results were aligned by frame number and used in later phase segmentation and feature extraction modules to construct ankle-, knee-, hip-, and pelvis-related time-series signals. This processing allowed human movement information to participate in key-event detection and phase-specific kinematic calculation on a unified video timeline and spatial reference. To improve trajectory continuity and reduce the influence of instantaneous detection errors, only the core keypoints directly related to hurdling analysis, including the bilateral shoulders, hips, knees, and ankles, were retained. When a keypoint confidence score was below 0.5, the corresponding coordinate was set to missing. Linear interpolation was then used to fill missing coordinates, with the maximum continuous interpolation length set to 8 frames; remaining missing values at the beginning or end of the sequence were filled using forward and backward filling. The same procedure was applied to the shoulder midpoint, pelvis midpoint, and center-of-mass-related variables to ensure complete time-series signals for event detection and feature extraction.

### 2.5. Variable Extraction

Hurdle detection results and two-dimensional markerless pose estimation results were fused using frame number as the common temporal index, constructing a unified data structure containing hurdle geometry, human pose coordinates, and video timing information. At this stage, only variables that could be directly extracted or calculated from frame-wise aligned data were retained, without including phase-dependent indicators that required phase segmentation.

The hurdle detection module output hurdle bounding-box coordinates, bar midpoint coordinates, post-reference position, and detection confidence, which were used to build an external spatial reference for subsequent movement analysis. The pose estimation module output the 17 body keypoints, keypoint confidence scores, and person bounding-box information. After frame-wise alignment, bilateral joint coordinates were used to calculate pelvis midpoint, shoulder midpoint, lower-limb segment lengths, and body-scale descriptive variables.

Based on bilateral keypoint coordinates, the pelvis midpoint and shoulder midpoint were first constructed as representative points of whole-body posture. The pelvis midpoint was defined as:

(1) $$x_{pelvis}\;=\;\frac{x_{left\_hip\;}+\;x_{right\_hip}}{2},\;{\;y}_{pelvis}\;=\;\frac{y_{left\_hip\;}+{\;y}_{right\_hip}}{2}\;$$

The shoulder midpoint was defined as:

(2) $$x_{shoulder}\;=\;\frac{x_{left\_shoulder}\;+\;x_{right\_shoulder}}{2},\;y_{shoulder}\;=\;\frac{y_{left\_shoulder\;}+\;y_{right\_shoulder}}{2}$$

The body center of mass was estimated as:

(3) $$p_{COM}\;=\;w_{u}p_{upper}\;+\;w_{t}p_{thigh}\;+\;w_{l}p_{lower}$$

Upper-body representative point was:

(4) $$w_{u}\;+\;w_{t}\;+\;w_{l}\;=\;1$$

Upper-body representative point was:

(5) $$p_{upper}\;=\;w_{s}p_{shoulder}\;+\;w_{h}p_{pelvis}$$

Weights used in the current implementation [21] were: $w_{u}=0.60$, $w_{t}=0.28$, $w_{l}=0.12$, $w_{s}=0.45$, and $w_{h}=0.55$.

The output variables are summarized in Table 2.

The hurdle geometry was further used to estimate the real hurdle height and construct the pixel-to-meter conversion scale:

(6) $$s=\frac{H_{real}}{H_{pixel}}$$

In addition, center-of-mass-related frame-wise variables were estimated from the fused pose data, and their time derivatives were calculated using the video frame rate. These frame-wise geometric variables formed the basic input for subsequent key-event detection, phase segmentation, and phase-specific kinematic analysis.

### 2.6. Event-Based Phase Segmentation

#### 2.6.1. Definition of Key Events

To segment the hurdle-clearance action, four key events were defined: takeoff touchdown, takeoff toe-off, swing-leg touchdown, and swing-leg toe-off. Takeoff touchdown refers to the instant at which the takeoff leg establishes support before the hurdle; takeoff toe-off refers to the instant at which the takeoff leg completes push-off and leaves the ground; swing-leg touchdown refers to the first ground contact of the lead/swing leg after hurdle clearance; and swing-leg toe-off refers to the instant at which the recovery leg leaves the ground after post-hurdle support. These four events correspond to key support and flight transitions during hurdle clearance and can therefore serve as temporal boundaries for phase segmentation.

#### 2.6.2. Definition of Three Phases

**Figure 5:**
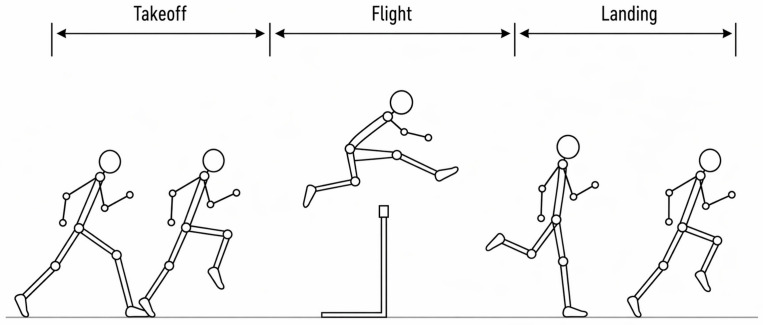
Definition of the three hurdle-clearance phases based on four key events.

Based on these key events, the hurdle-clearance action was divided into three phases: takeoff, flight, and landing (Figure 5). The takeoff phase was defined as the interval from takeoff touchdown to takeoff toe-off; the flight phase was defined as the interval from takeoff toe-off to swing-leg touchdown; and the landing phase was defined as the interval from swing-leg touchdown to swing-leg toe-off [1,4,22]. The fused frame-wise data were assigned phase labels according to the key-event positions on the timeline to support subsequent phase-specific feature extraction and validation analysis.

#### 2.6.3. Rule-Based Event Detection Procedure

For event-driven phase segmentation, the same four key events were detected: takeoff touchdown, takeoff toe-off, swing-leg touchdown, and recovery toe-off. These events represent the establishment of takeoff support, termination of takeoff support, first post-hurdle support, and termination of recovery support, respectively, and serve as temporal boundaries for the takeoff, flight, and landing phases. Before event detection, the two-dimensional ankle and knee coordinate sequences were smoothed to reduce single-frame jitter. Specifically, centered moving averages with a 5-frame window were applied to the horizontal and vertical coordinates of the bilateral ankles and knees, and the knee angle sequence was smoothed using the same 5-frame window. Velocity and acceleration signals were then calculated from the smoothed ankle vertical trajectories, and key events were detected by combining bilateral ankle vertical differences, knee angle changes, and the spatial relationship between the athlete and the hurdle. This procedure was intended to improve event detection stability and physiological plausibility. The event definitions and decision rules are shown in Table 3.

### 2.7. Phase-Specific Kinematic Feature Extraction

After key-event detection and phase segmentation, phase-specific kinematic features were extracted to describe technical performance during the takeoff, flight, and landing phases. Feature extraction was based on fused frame-wise geometric variables, hurdle reference information, and key-event frames, and the relevant indicators were mapped to the corresponding movement intervals according to phase boundaries.

The takeoff phase mainly included variables reflecting pre-hurdle support and push-off, such as takeoff distance, takeoff support time, knee angle at takeoff touchdown, knee angle at takeoff toe-off, and trunk angle at takeoff toe-off. The flight phase mainly included variables reflecting airborne clearance and posture control, such as flight time, center-of-mass rise, lead-leg knee angle at hurdle crossing, and trunk angle at hurdle crossing. The landing phase mainly included variables reflecting post-hurdle absorption and recovery, such as landing distance, knee angle at landing, shank angle at landing, trunk angle at landing, and recovery time. Through this procedure, the system generated distance-, time-, and angle-related technical indicators with explicit phase attributes from the complete hurdle-clearance action.

All angle-related variables were calculated from two-dimensional keypoint coordinates. For a joint angle formed by three points A, B, and C, where B is the vertex, the angle θ was defined as:

(7) $$\theta ={cos}^{-1}\left(\frac{\vec{BA}\;\cdot \vec{BC}}{|\vec{BA}||\vec{BC}|}\right)$$

where the two vectors denote the directions from the vertex to the adjacent keypoints. This method was used to calculate knee angles, hip angles, and trunk inclination variables. Through these procedures, this study achieved structured extraction of phase-specific kinematic features from hurdle clearance and provided quantitative outputs for subsequent technical analysis and method validation.

### 2.8. Validation Experiments

#### 2.8.1. Event Detection Validation

To validate the accuracy of automatic phase segmentation, four key events were first manually annotated: takeoff touchdown, takeoff toe-off, swing-leg touchdown, and swing-leg toe-off. Manual annotation was performed frame by frame using the original video frame sequence, and the frame number corresponding to each key event was recorded. Automatic detection results were compared with manual annotations for the same videos. For valid samples, the mean absolute error (MAE) and root mean square error (RMSE) of each key event were calculated and reported in both frames and seconds. Frame error was calculated as the difference between the automatically detected frame and the manually annotated frame, and time error was obtained by converting frame error into seconds using the video frame rate. These metrics provided a quantitative evaluation of temporal event localization accuracy.

#### 2.8.2. Versus Temporal Baseline Models

To further evaluate the proposed rule-based event detection method, two temporal baseline models were implemented: a long short-term memory network (LSTM) and a temporal convolutional network (TCN). These models were used as data-driven baselines for frame-wise phase classification and subsequent event-frame localization.

The input features for the LSTM and TCN models were extracted from the same frame-wise feature tables used in the proposed pipeline. These features included two-dimensional body keypoint coordinates, keypoint confidence scores, hurdle position, bar midpoint, post-reference position, pelvis and shoulder midpoint coordinates, estimated center-of-mass coordinates, leg-length-related variables, center-of-mass velocities, and hurdle-relative spatial variables. Coordinate features were normalized by image width or height, and numerical features were standardized using the mean and standard deviation calculated from the training videos in each fold.

Manual event annotations were used to generate frame-wise phase labels. Frames from takeoff-leg touchdown to takeoff-leg toe-off were labeled as the takeoff phase, frames from takeoff-leg toe-off to swing-leg touchdown were labeled as the flight phase, and frames from swing-leg touchdown to swing-leg toe-off were labeled as the landing phase. Frames outside these intervals were labeled as outside. After frame-wise phase prediction, event frames were inferred from the predicted phase transitions and compared with manual annotations.

Model evaluation was conducted using video-level five-fold cross-validation. In each fold, all frames from the same video were assigned exclusively to either the training set or the test set to prevent frame-level data leakage. The LSTM baseline consisted of a two-layer bidirectional LSTM with 64 hidden units and a dropout rate of 0.2, followed by a linear classification layer. The TCN baseline consisted of a one-dimensional temporal convolutional network with four residual temporal convolution blocks using dilation factors of 1, 2, 4, and 8. Both models used four output classes: outside, takeoff, flight, and landing.

The models were trained for a maximum of 120 epochs using the AdamW optimizer, with early stopping applied when the training loss did not improve for 18 consecutive epochs. Class-weighted cross-entropy loss was used to reduce the influence of phase-length imbalance. The evaluation metrics included phase classification accuracy, macro-F1 score, event localization MAE, RMSE, and Max AE. To examine the effect of potentially ambiguous samples, a clean subset analysis was also conducted after excluding one outlier video and four videos marked as requiring rule-based correction or estimation.

#### 2.8.3. Kinematic Variable Validation

To verify the agreement of the core kinematic variables extracted by the system, a kinematic variable validation experiment was conducted. Representative indicators were selected, including takeoff distance, flight time, landing distance, lead-leg knee angle at hurdle crossing, and trunk angle at hurdle crossing. For selected video samples, reference values were obtained by manual frame-by-frame measurement or auxiliary software measurement and compared with the automatic outputs of the system.

#### 2.8.4. Stability Analysis

Agreement between manually recalculated and system-derived core kinematic variables was evaluated using MAE, RMSE, intraclass correlation coefficients (ICC(A,1)), and Bland–Altman bias and limits of agreement (LOA).

#### 2.8.5. Pose Estimation

Bilateral shoulder, hip, knee, and ankle keypoints were manually annotated to evaluate pose-estimation accuracy at two levels [17]. First, key-event frames and hurdle-crossing frames were annotated because many phase-specific variables were calculated directly from joint coordinates at these biomechanically important instants. Second, frame-wise annotation was performed on all 37 complete hurdle-clearance videos to assess the temporal stability of keypoint localization across the full movement sequence. For frame-wise evaluation, the annotated frames were grouped into five intervals: start, takeoff, flight, landing, and continuation. The takeoff, flight, and landing intervals were defined by adjacent key events, whereas the start and continuation intervals referred to frames before takeoff-leg touchdown and after recovery-leg toe-off, respectively. Manual reference coordinates were compared with system outputs using RMSE and percentage of correct keypoints (PCK).

RMSE was used to quantify the average deviation between system keypoint coordinates and manual reference coordinates. Let the manually annotated coordinates of a keypoint in the i-th sample be ($x_{i}^{m}$, $y_{i}^{m}$) and the system-generated coordinates be ($x_{i}^{a}$, $y_{i}^{a}$). The two-dimensional Euclidean distance error is defined as:

(8) $$e_{i}=\sqrt{(x_{i}^{a}-x_{i}^{m}{)}^{2}+(y_{i}^{a}-y_{i}^{m}{)}^{2}}$$

The RMSE was defined as:

(9) $$RMSE=\sqrt{\frac{1}{N}\sum_{i=1}^{N}\;e_{i}^{2}}$$

where N denotes the number of keypoint samples included in the analysis. RMSE was expressed in pixels and reflected the overall localization error of system outputs relative to manual reference annotations. A smaller RMSE indicates closer agreement with manual annotation.

The percentage of correct keypoints (PCK) was used to evaluate whether system outputs fell within an acceptable error range. In this study, a distance-threshold-based criterion was used: when the two-dimensional Euclidean distance between the system output keypoint and the manual reference keypoint was smaller than a predefined threshold T, the keypoint was considered correctly detected. PCK was defined as:

(10) $$PCK=\frac{N_{correct}}{N}\times 100\%$$

where $N_{correct}$ denotes the number of keypoints with errors below the threshold *T* and *N* denotes the total number of keypoints. PCK reflects the proportion of successfully detected keypoints under a given tolerance; higher values indicate more stable keypoint localization and better agreement with manual reference annotation. Because all videos were recorded with a fixed side-view setup, normalized to the same resolution, and showed relatively limited changes in athlete scale, a strict PCK@0.02 criterion was used to constrain keypoint localization error within a small range and better satisfy the accuracy requirements for subsequent phase segmentation and kinematic feature extraction.

## 3. Results

### 3.1. Sample Characteristics

A total of 37 monocular side-view hurdle-clearance videos were collected and uniformly preprocessed before entering the analysis pipeline. The preprocessed videos were normalized to 1280 × 720 pixels, with a frame rate of approximately 60 fps, and each video covered one complete hurdle-clearance cycle. Based on video quality and completion of the automatic analysis workflow, only samples that successfully completed hurdle detection, human pose estimation, key-event detection, and phase segmentation were included in the results analysis.

Finally, 37 videos were included in the subsequent analysis. All included samples completed hurdle detection, pose estimation, key-event detection, and phase segmentation and served as the basis for analyzing hurdle detection outputs, pose estimation results, phase segmentation results, and validation experiments.

### 3.2. Hurdle Detection

**Figure 6:**
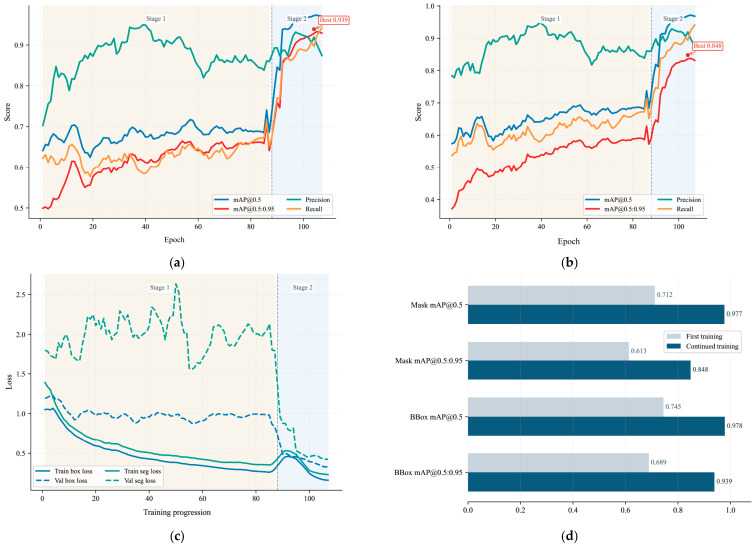
Training curves and representative performance of the hurdle detection model. (**a**) Changes in bounding-box detection performance. (**b**) Changes in mask segmentation performance. (**c**) Convergence process of training losses. (**d**) Comparison of key performance indicators before and after continued training.

After two-stage training, the hurdle detection model showed improved detection and segmentation performance compared with the first-stage model (Figure 6). Because the main purpose of this study was to verify the feasibility of the complete analysis pipeline rather than to compare object detection models, the training curves and representative visual outputs were used to confirm stable hurdle localization for subsequent kinematic analysis. The improved model provided more consistent bounding-box localization and segmentation masks, supporting subsequent extraction of hurdle reference points.

As shown in Table 4, the model achieved bounding-box detection performance of Precision = 0.969, Recall = 0.958, mAP@0.5 = 0.992, and mAP@0.5:0.95 = 0.942. For segmentation masks, the corresponding values were Precision = 0.955, Recall = 0.945, mAP@0.5 = 0.971, and mAP@0.5:0.95 = 0.755. These results indicate that the model could accurately localize and segment the hurdle target and showed good stability and generalization performance on the independent test set. Compared with the bounding-box results, the segmentation mask performance was slightly lower under stricter IoU thresholds, suggesting that further improvement is still needed in delineating fine object-boundary details.

**Figure 7:**
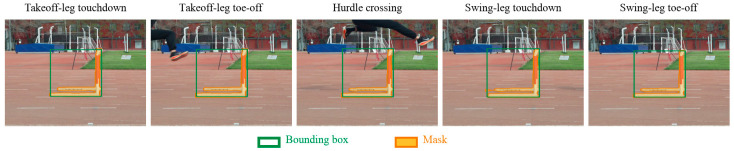
Representative hurdle detection results and reference point extraction.

In the included video samples, the hurdle could generally be detected continuously throughout the complete clearance cycle. Based on detection results, the system output frame-wise hurdle bounding-box coordinates, bar midpoint coordinates, and post-reference positions. The bar midpoint was mainly used to determine the relationship between human keypoints and the hurdle during clearance, while the post-reference position was used for calculating spatial indicators, such as takeoff distance and landing distance. Representative results showed that the hurdle bounding box and reference points maintained good spatial consistency at key moments before takeoff, during clearance, and after landing (Figure 7).

Overall, the hurdle detection module not only achieved stable object detection and segmentation but also provided an external spatial reference for subsequent key-event detection and phase-specific kinematic feature extraction. This indicates that extracting hurdle spatial reference information from monocular side-view video is feasible in the present study setting.

### 3.3. Pose Estimation Outputs

**Figure 8:**
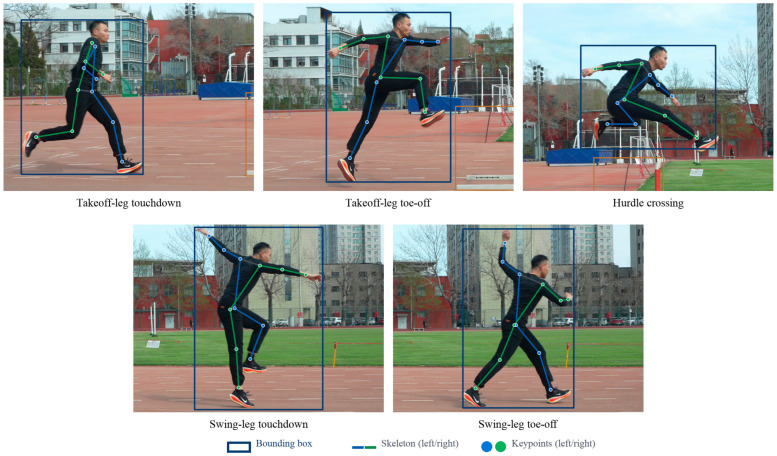
Representative human pose estimation outputs at key moments of hurdle clearance.

The human pose estimation module output frame-wise two-dimensional keypoint results in the included monocular side-view videos and completed person bounding-box localization and keypoint tracking for the target athlete. Representative results showed that the bilateral shoulders, hips, knees, and ankles, which are closely related to hurdle phase segmentation and kinematic feature extraction, could be identified relatively stably at typical moments before takeoff, during hurdle crossing, and after landing (Figure 8). To further evaluate the usability of the keypoint results in this task, bilateral shoulders, hips, knees, and ankles were manually annotated at key-event frames and hurdle-crossing frames, and the manual coordinates were compared with system outputs. Evaluation metrics included RMSE and the PCK.

Keypoint localization accuracy was first evaluated at the key frames used for event definition and phase-specific variable extraction (Table 5). This key-frame-based evaluation was necessary because several kinematic variables were calculated directly from joint coordinates at discrete biomechanical instants, including takeoff-leg touchdown, takeoff-leg toe-off, the hurdle-crossing instant, swing-leg touchdown, and swing-leg toe-off. Therefore, the localization accuracy of relevant keypoints at these frames directly affects event detection, phase labeling, and the calculation of variables such as takeoff distance, landing distance, knee angle, trunk angle, and center-of-mass-related indicators.

The keypoint annotation results at these key frames showed high consistency (Table 5). Across all keypoint samples, the overall RMSE was 8.25 px, and the overall PCK was 97.64%, indicating good agreement between system outputs and manual reference annotations in most cases. Keypoint-specific results showed that proximal body points were localized more accurately. The overall RMSE values for the left and right shoulders were 0.95 px and 1.00 px, with corresponding PCK values of 98.92% and 98.38%; the overall RMSE values for the left and right hips were 2.14 px and 1.86 px. Knee points also performed relatively stably, with RMSE values of 1.88 px for the right knee and 7.07 px for the left knee. In contrast, ankle errors were larger, with overall RMSE values of 12.70 px for the left ankle and 17.87 px for the right ankle, suggesting that distal lower-limb keypoints are more susceptible to errors during fast leg swing and occlusion.

Event-specific results showed that keypoint localization was most stable at the takeoff-related and landing-touchdown frames. The overall RMSE and PCK were 0.61 px and 100.00% at takeoff-leg touchdown, 1.59 px and 97.30% at takeoff-leg toe-off, and 1.52 px and 97.97% at swing-leg touchdown. These results indicate that the keypoints used to determine the main support-transition events were detected with high accuracy. In contrast, the overall RMSE increased to 13.83 px at hurdle crossing and 11.99 px at swing-leg toe-off. The largest error occurred for the right ankle at hurdle crossing (RMSE = 38.82 px, PCK = 83.78%), and the left ankle at swing-leg toe-off also showed a relatively high error (RMSE = 28.40 px).

These findings suggest that the pose estimation module provided sufficiently accurate keypoint localization at the biomechanically important frames required for phase segmentation and phase-specific kinematic feature extraction. The shoulders, hips, and knees showed stable localization across key events, whereas errors were mainly concentrated in ankle keypoints during hurdle crossing and late post-hurdle recovery. This error pattern is consistent with the high movement speed and frequent lower-limb overlap observed during hurdle clearance.

To further examine pose-estimation performance over the complete hurdle-clearance process, frame-wise accuracy was assessed across all annotated video frames (Table 6). Unlike the key-event-based evaluation in Table 5, which focused on discrete biomechanical instants used for event definition and variable extraction, this analysis evaluated whether the pose estimation module maintained stable localization performance throughout the continuous movement sequence.

The frame-wise results showed that proximal keypoints remained highly stable across the start, takeoff, flight, landing, and continuation intervals. Shoulder and hip keypoints generally showed very small RMSE values and PCK values close to 100%, indicating consistent localization throughout the full sequence. In contrast, larger errors were mainly observed in distal lower-limb keypoints during the flight and landing intervals. The left ankle showed the largest error during the flight interval, with an RMSE of 29.06 px and a PCK of 91.98%. During the landing interval, increased errors were also observed for the left knee, left ankle, and right ankle, with RMSE values of 10.30 px, 13.90 px, and 14.37 px, respectively.

These frame-wise findings indicate that the pose estimation module remained stable across most of the complete hurdle-clearance process, while the main errors occurred in distal lower-limb keypoints during high-speed leg swing, hurdle clearance, and landing. This pattern is consistent with the key-event-based results and supports the use of the pose estimation outputs for subsequent event detection, phase segmentation, and phase-specific kinematic feature extraction.

### 3.4. Phase Segmentation Results

The comparison with temporal baseline models is shown in Table 7. For all 37 video samples, the rule-based method achieved the lowest overall event localization error, with an MAE of 0.74 frames and an RMSE of 1.55 frames. In comparison, the LSTM and TCN baselines showed higher overall errors, with MAE/RMSE values of 1.87/4.31 frames and 1.70/4.21 frames, respectively.

Event-wise results showed that the rule-based method performed particularly well for takeoff-related events. For takeoff-leg touchdown and takeoff-leg toe-off, the rule-based method achieved MAE/RMSE/Max AE values of 0.16/0.40/1 frames and 0.62/0.85/2 frames, respectively, which were lower than those of both temporal baseline models. For swing-leg touchdown, the three methods showed relatively close MAE values, although the TCN baseline produced a lower RMSE and Max AE than the rule-based method. For swing-leg toe-off, the TCN baseline achieved the lowest MAE and RMSE, suggesting that temporal models may have potential advantages for landing-related events.

Because the rule-based results showed a mean error of only 1.49 frames for the swing-leg toe-off event but the maximum error reached 12 frames, we suspected that an extreme deviation occurred in one specific sample. Therefore, this sample was excluded, and the models were trained and evaluated again. After excluding one special case, the comparison between the rule-based method and temporal baseline models was repeated using 36 video samples (Table 8). The rule-based method achieved the lowest overall MAE, with an event localization error of 0.63 frames, compared with 1.75 frames for LSTM and 1.66 frames for TCN. The rule-based method also showed the lowest overall RMSE (1.14 frames), whereas LSTM and TCN showed RMSE values of 4.27 and 4.25 frames, respectively.

Event-wise results showed that the rule-based method remained most accurate for takeoff-related events. For takeoff-leg touchdown, the rule-based method achieved an MAE/RMSE/Max AE of 0.19/0.44/1 frames, which was lower than both LSTM and TCN. For takeoff-leg toe-off, the rule-based method also showed lower error than the temporal baselines, with an MAE/RMSE/Max AE of 0.61/0.85/2 frames. For swing-leg touchdown and swing-leg toe-off, the temporal models showed competitive performance. In particular, TCN achieved lower RMSE and Max AE for swing-leg touchdown, and both LSTM and TCN showed lower Max AE for swing-leg toe-off.

Overall, these results suggest that, after excluding one special case, the rule-based method still provided the most stable overall event localization and remained especially robust for takeoff-related events. At the same time, the temporal baseline models, particularly TCN, showed potential advantages for landing-related events, indicating that data-driven temporal models may be useful for improving post-hurdle event detection when larger annotated datasets become available.

### 3.5. Phase-Specific Kinematic Variables

#### 3.5.1. Kinematic Variable Output Results

After key-event detection and phase segmentation, the system further output core kinematic variables for the takeoff, flight, and landing phases. Table 9 summarizes the core phase-specific kinematic variables for the included samples. Overall, variables from each phase could be output stably and showed a certain degree of between-sample variability, reflecting individual differences in hurdling technique. Takeoff variables mainly reflected pre-hurdle support and push-off characteristics, flight variables mainly reflected time allocation and airborne posture control, and landing variables mainly reflected post-hurdle absorption and recovery. These results show that the proposed method can not only segment movement phases but also produce phase-specific technical indicators with biomechanical meaning.

**Figure 9:**
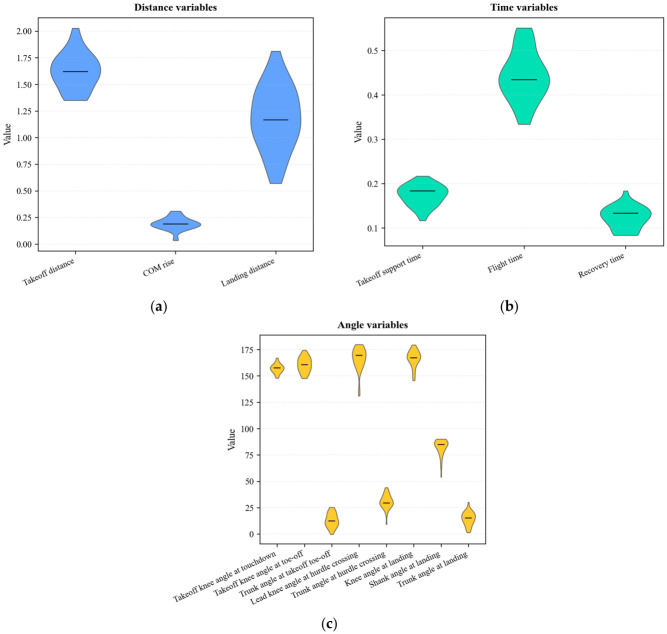
Representative phase-specific kinematic variable outputs. (**a**) Distance-related variables. (**b**) Time-related variables. (**c**) Angle-related variables.

Figure 9 shows the phase-specific variable outputs of a representative sample. The results indicate that monocular side-view video combined with markerless pose estimation can provide a phase-specific quantitative description of hurdle clearance and support subsequent variable accuracy testing and technical analysis.

#### 3.5.2. Accuracy Validation of Core Kinematic Variables

Using manually annotated key-event frame coordinates, selected variables were recalculated and compared with the automatic system outputs on a video-by-video basis. MAE, RMSE, ICC, Bland–Altman bias, and LOA were used to evaluate consistency (Table 10).

Distance variables showed the highest agreement. The MAE values for takeoff distance and landing distance were 0.0014 m and 0.0013 m, respectively, and their RMSE values were 0.0017 m and 0.0016 m. Both variables showed ICC(A,1) values of 1.000, with negligible bias and very narrow limits of agreement. Among time variables, takeoff support time and flight time showed ICC(A,1) values of 0.738 and 0.864, respectively, whereas recovery time showed a markedly lower ICC(A,1) of 0.211. These results indicate that time-related variables, especially recovery time, were more sensitive to event-frame deviation than distance-related variables.

Angle variables also showed good overall agreement. The ICC(A,1) values for takeoff-leg knee angle at touchdown, takeoff-leg knee angle at toe-off, lead-leg knee angle at hurdle crossing, knee angle at landing, shank angle at landing, trunk angle at takeoff toe-off, and trunk angle at landing were 0.985, 0.973, 0.992, 0.990, 0.998, 0.990, and 0.981, respectively, indicating high agreement with the manual reference. In contrast, trunk angle at hurdle crossing showed a relatively lower ICC(A,1) of 0.872, with a bias of −1.1752° and wider limits of agreement (−7.4601 to 5.1098°). Center-of-mass rise showed an ICC(A,1) of 0.865 and a negative bias of −0.0209 m, indicating that the system slightly underestimated this variable relative to the manual reference.

## 4. Discussion

### 4.1. Summary

This study developed a hurdle action analysis framework based on the RTMPose human pose estimation model and the YOLO26 object detection algorithm. The framework integrated hurdle detection and segmentation, human keypoint extraction, key-event detection, phase segmentation, and phase-specific kinematic variable output. The hurdle detection module was evaluated using an independent test set, and the event-based phase segmentation method was further compared with temporal baseline models (LSTM and TCN). In addition, both key-event-based and frame-wise pose-estimation validations were conducted to assess keypoint localization accuracy at biomechanically important instants and across the complete hurdle-clearance sequence. Together, these steps formed a complete automatic analysis pipeline for hurdle clearance, allowing key structural features of the clearance movement to be identified from ordinary training videos.

The proposed event-driven phase segmentation method showed good feasibility. The system established phase boundaries based on four key events, namely takeoff touchdown, takeoff toe-off, swing-leg touchdown, and swing-leg toe-off, and further output core technical indicators for each phase. Validation results showed small overall errors between automatic event detection and manual references, with a mean absolute error of approximately 0.74 frames, corresponding to approximately 0.012 s. Compared with the LSTM and TCN temporal baselines, the rule-based method showed lower overall event-localization error, particularly for takeoff-related events, although the temporal models showed competitive performance for some landing-related events. This indicates that the method can provide accurate and interpretable phase segmentation for subsequent phase-specific variable extraction.

Pose estimation results also supported the applicability of the workflow. Core keypoints including the bilateral shoulders, hips, knees, and ankles showed good agreement with manual annotations at key biomechanical frames, with an overall RMSE of 8.25 px and a PCK of 97.64%. Frame-wise validation further showed that keypoint localization remained stable across the complete hurdle-clearance sequence, especially for proximal keypoints such as the shoulders and hips. Shoulder, hip, and knee keypoints were relatively stable, whereas larger errors were mainly concentrated at ankle keypoints during hurdle crossing, swing-leg toe-off, and the flight and landing intervals. These findings indicate that current markerless pose estimation can provide usable input for hurdle phase segmentation and most kinematic variable calculations, although distal lower-limb keypoints remain the main source of localization error.

The hurdle detection module showed good performance on the independent test set, indicating that the hurdle could be reliably localized and segmented in independently recorded videos. This enabled the extraction of hurdle-relative spatial references, including hurdle position, bar midpoint, and post-reference points. For phase-specific variable output, the system extracted relevant indicators stably. Compared with manual reference results, distance variables and most angle variables showed good absolute agreement, indicating that integrating hurdle spatial references with human keypoints can transform ordinary two-dimensional video into hurdle-specific technical indicators. Unlike simple human pose estimation, the present study introduced hurdle position, bar midpoint, and post-reference points, allowing the athlete’s movement to be interpreted within a hurdle-relative spatial framework. This represents a distinctive feature of the proposed method.

### 4.2. Practical Feasibility and Field Applicability

The results indicate that monocular side-view video analysis of hurdle clearance has good low-cost field applicability. Compared with traditional three-dimensional motion capture systems, multi-camera high-speed video systems, or inertial sensor measurement schemes, the data collection equipment required in this study is easier to obtain. Videos can be recorded using ordinary smartphones, cameras, or other common imaging devices, without attaching markers or wearable sensors to the athlete. This reduces interference with training and is better suited for routine practice environments.

The hardware requirements for data analysis were also reduced. The software workflow in this study was run on an Apple laptop computer in a CPU-only environment, with each video requiring approximately one minute for analysis, and did not depend on a dedicated NVIDIA GPU or high-performance workstation. Although deep-learning-based visual models are often considered computationally demanding, this task showed that ordinary personal computers can complete hurdle detection, pose estimation, phase segmentation, and kinematic variable extraction for single hurdle videos when the workflow is properly designed. The current workflow was implemented on a MacBook Air with an M4 chip, 16 GB of memory, and macOS. A single hurdle-clearance video required approximately 45 s for complete analysis, including model loading, hurdle detection, pose estimation, phase segmentation, and variable extraction. Because model loading is required only once during continuous processing, batch analysis is expected to further reduce the average processing time per video. Therefore, the system is more suitable for offline or near-session feedback than for real-time use. Such hardware conditions are more accessible for grassroots training, school physical education, and ordinary sports teams.

The main requirement of the method lies in video acquisition quality. As long as the camera is positioned approximately side-on to the hurdling action, the athlete and hurdle are kept in the same main motion plane, and the video fully records takeoff, flight, and landing, the system can obtain relatively stable input. The image should also be sufficiently clear, with minimal severe occlusion, motion blur, and camera shake, so that the hurdle structure and body keypoints can be detected reliably. In this sense, the method mainly requires users to follow basic video recording standards.

From a practical perspective, this low-cost feature gives the proposed method strong potential for broader application. Coaches or researchers can record hurdle-clearance videos during routine training using common imaging devices and complete the subsequent analysis on an ordinary laptop computer, obtaining event-specific indicators such as takeoff distance, flight time, landing distance, trunk angle, knee angle, and center-of-mass changes. This approach reduces dependence on specialized equipment, complex field setup, and manual frame-by-frame measurement, bringing kinematic analysis closer to actual training needs.

Therefore, the proposed method has advantages in equipment accessibility, operational convenience, and field applicability. Its aim is not to replace laboratory-level high-precision measurement in all contexts but to provide a low-cost, low-interference, repeatable technical analysis solution for hurdling training and teaching within an acceptable accuracy range.

### 4.3. Accuracy and Error Characteristics of the Proposed Method

#### 4.3.1. Acceptability of Overall Accuracy

The overall accuracy of the system was satisfactory. As shown in Table 5, the overall RMSE of human keypoint localization was 8.25 px, and the PCK was 97.64%, indicating that system outputs agreed well with manual reference annotations in most cases. Desai et al. used PCK and PDJ to evaluate keypoint detection accuracy in yoga pose estimation and reported that a PCK above 90% can support subsequent posture analysis [23]. In addition, relevant reviews have reported that recent two-dimensional pose estimation methods often achieve detection rates above 90% for major body joints, suggesting that a high PCK level can be considered an important indicator of keypoint usability [24,25]. Therefore, the present keypoint localization results provide a suitable input basis for subsequent phase segmentation and phase-specific kinematic variable calculation.

This was also reflected in the subsequent event detection and variable extraction results. Table 7 shows that the overall key-event detection error was small, and Table 9 shows that most core kinematic variables maintained good absolute agreement with manually recalculated values, especially distance variables and most lower-limb angle variables. These findings indicate that the proposed keypoint detection, key-event detection, and phase-specific variable extraction workflow is generally reliable and can meet the basic requirements of automatic hurdle action analysis under the present recording conditions.

#### 4.3.2. Influence of Larger Errors on Biomechanical Variables

However, accuracy was not uniform across all keypoints and phases. Table 5 shows that errors were mainly concentrated at the ankle, especially at hurdle crossing and swing-leg toe-off. Previous studies have shown that distal joints are more susceptible to occlusion, local blur, and rapid movement changes than proximal joints, and therefore, their accuracy tends to decrease [17,19,23].

These keypoint-level errors may affect phase-specific biomechanical variables differently. Correspondingly, swing-leg touchdown and swing-leg toe-off showed larger errors in Table 7, while recovery time, trunk angle at hurdle crossing, and center-of-mass rise showed relatively lower agreement or wider limits of agreement in Table 9. These results suggest that the main current errors are concentrated in distal lower-limb keypoints and landing-phase-related indicators.

**Figure 10:**
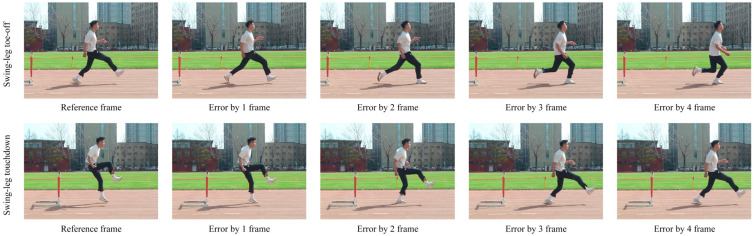
Frame-by-frame visual comparison of 1–4 frame event-detection errors for swing-leg touchdown and swing-leg toe-off.

Figure 10 displays representative 1–4 frame offsets from the manually identified reference for swing-leg touchdown and toe-off.

A single-frame offset produced only subtle postural changes that were difficult to pick up visually; distance and angular variables remained largely unaffected. Hence, errors of ~1 frame are acceptable for general phase segmentation and routine field-based feedback.

With a two-frame discrepancy, changes in joint configuration grew more noticeable. Upper-body posture stayed relatively stable, yet lower-limb angles—especially the knee—shifted more markedly because of the rapid transitions of the swing and support legs during landing. Distance estimates also drifted; landing position could vary by roughly half a foot length. Two-frame errors may therefore still serve coarse technical feedback, but they introduce meaningful uncertainty in variables that hinge on knee configuration or foot-contact timing.

Once deviations reached three frames, the differences became visually obvious. Trunk orientation, lower-limb alignment, and foot–ground relationship all altered enough to compromise both angular and distance-based metrics. Such errors should be treated with caution, particularly for landing-phase indicators and any measure derived from distal lower-limb keypoints. This sensitivity explains why mistimed swing-leg touchdown or toe-off exerts a greater influence on recovery time, landing-related timing, knee angle, and similar phase-specific indicators than comparable errors occurring during steadier movement periods.

When interpreted with Table 7, the practical impact of these frame-level errors appears limited. The mean errors for the latter two events were 0.81 and 1.49 frames, respectively. Within this range, upper-body posture, distance-related variables, and center-of-mass-related variables showed only minor changes. In contrast, lower-limb angle variables, especially those involving rapid knee and ankle motion, were more sensitive to small temporal shifts and should be interpreted cautiously.

#### 4.3.3. Sources of Error and Potential Improvements

**Figure 11:**
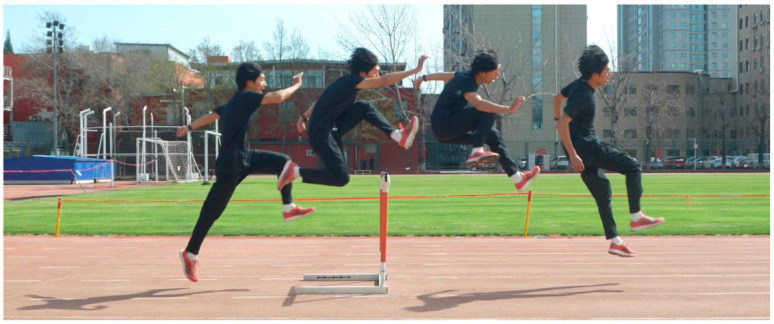
Example of an atypical landing pattern causing large event-detection error.

A notable outlier in Table 7 further illustrates the practical boundary of the proposed framework. The maximum 12-frame error occurred in a participant who had not yet fully mastered the hurdle-clearance technique. During the post-hurdle landing phase, this participant adopted a movement strategy similar to jumping over the hurdle, resulting in an almost simultaneous two-foot landing posture (Figure 11). This atypical landing pattern substantially violated the expected support–flight–support sequence assumed by the rule-based segmentation strategy, leading to a large deviation from the manually identified event frame.

In this specific case, the LSTM and TCN baselines showed smaller errors than the rule-based method, with errors of approximately five frames. However, even this level of error remains too large for reliable extraction of phase-specific biomechanical indicators. Therefore, when an athlete shows clearly atypical or technically immature hurdle-clearance patterns, the proposed framework should not be used as the primary basis for biomechanical interpretation. In applied coaching, the first priority should be to correct the visibly incorrect movement pattern and help the athlete establish a more stable hurdle-clearance technique. Once the athlete reaches a level at which major technical errors are no longer obvious through visual observation, biomechanical indicators derived from this framework may provide more meaningful support for identifying subtle technical deficiencies and guiding further refinement.

**Figure 12:**
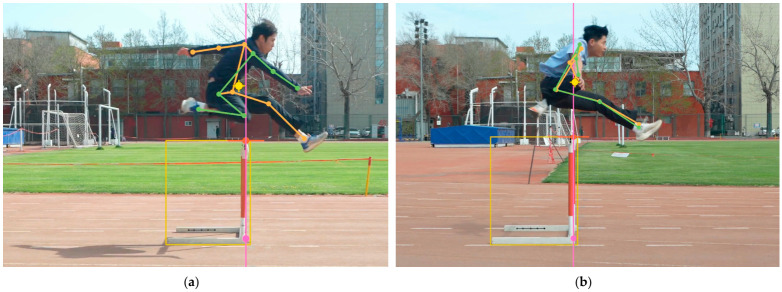
Representative examples of ankle localization errors under different image conditions. (**a**) Keypoint detection under strong direct lighting combined with black trousers. (**b**) Keypoint detection under diffuse lighting combined with black trousers.

More specifically, the most prominent weak points were the right ankle at the hurdle-crossing instant and the right hip at swing-leg touchdown (Table 5). The relatively low PCK of the right ankle at hurdle crossing has a clear image-based explanation. In the corresponding frames, under even but weak lighting, the athlete wore black trousers, the takeoff leg was markedly folded during hurdle crossing, and the two legs overlapped closely in the image. This made the boundaries between the left and right lower limbs difficult to distinguish, especially around the ankle region. Under these conditions, the two-dimensional pose estimation model had difficulty locating the right ankle accurately, increasing the error at that instant (Figure 12). The representative images show that even black trousers can have clear boundaries under sunlight, whereas under cloudy conditions, motion blur in the foot region and indistinct trouser boundaries jointly led to right ankle localization failure. Such occasional errors may be reduced by increasing shutter speed and reducing motion blur during recording.

In contrast, the relatively low PCK of the right hip at swing-leg touchdown was less visually obvious. One possible explanation is that the hip joint is not an externally visible anatomical point with a clear image boundary but is indirectly estimated from the connection region between the trunk and thigh. Swing-leg touchdown occurs during rapid post-hurdle support transition, when pelvis posture, trunk inclination, and bilateral leg positions change quickly. As a result, the contour stability of the hip region may be reduced. This suggests that the right hip error at this stage may arise more from local pelvic configuration changes and uncertainty in joint-center estimation under two-dimensional projection than from clear occlusion. Although the right hip PCK at this stage was slightly below 90%, the RMSE of 3.38 indicates that the overall positional deviation remained small and acceptable.

### 4.4. Role of Hurdle Detection in Hurdle-Specific Spatial Modeling and Variable Extraction

An important feature of this study is the integration of a hurdle detection module in addition to human pose estimation. Many core indicators in hurdling technique analysis are variables describing the athlete’s spatial relationship with the hurdle. Previous hurdling kinematic studies have commonly examined takeoff distance, landing distance, the trajectory of the body center of mass during clearance, the position of the center of mass relative to the hurdle, and spatiotemporal characteristics during takeoff and landing [26,27]. Iwasaki et al. defined takeoff distance in a 110 m hurdle race as the horizontal distance from the foot to the hurdle at takeoff and landing distance as the horizontal distance from the foot to the hurdle at landing [28]. General obstacle-crossing studies have also emphasized the importance of foot-obstacle distance, clearance height, and obstacle height for movement control [29,30]. Together, these studies indicate that technical evaluation of hurdle clearance must be based on the spatial relationship between the body and the external obstacle.

Therefore, obtaining only shoulder, hip, knee, and ankle coordinates is insufficient for fully describing the event-specific technical characteristics of hurdle clearance. Human pose estimation can provide joint locations and limb posture information, but without hurdle position, hurdle height scale, and bar reference points, the system cannot determine whether the takeoff point is appropriate, whether the landing position facilitates post-hurdle running, or whether the center-of-mass clearance over the hurdle is economical. Krzeszowski et al. estimated hurdle-clearance parameters using a monocular human motion tracking method, demonstrating the feasibility of low-cost video methods for hurdling kinematic analysis [31]. Building on this idea, the present study further treated the hurdle as an automatically detected object in the analysis workflow, making it a spatial reference for kinematic variable calculation rather than merely a fixed background object.

The inclusion of hurdle detection allowed the system to convert human keypoints, which originally had only image-coordinate meaning, into hurdle-relative kinematic variables. For example, the system calculated takeoff distance from the relationship between the takeoff-leg touchdown point and the hurdle post, calculated landing distance from the relationship between the swing-leg touchdown point and the hurdle, evaluated the relative relationship between human keypoints and the hurdle at the crossing instant using the bar position, and estimated center-of-mass clearance and center-of-mass rise using the hurdle height scale. Thus, the system can not only determine where human keypoints are located but also explain how the athlete completes takeoff, clearance, and landing relative to the hurdle.

Accordingly, the hurdle detection module is not merely a visual front-end in this study but a key component for event-specific hurdling analysis. It expands the information available from simple human pose estimation in hurdling scenarios and enables monocular video analysis to automatically output technical indicators closer to those used in traditional hurdling kinematic research. This design improves the interpretability of the system for hurdle-clearance technique and provides clearer spatial evidence for subsequent training feedback and technical evaluation.

### 4.5. Scope and Future Development of the Proposed Framework

#### 4.5.1. Two-Dimensional Measurement Scope

The present results indicate that the proposed framework is feasible for field-based hurdle technique analysis, but it should be interpreted as an auxiliary two-dimensional assessment tool rather than a fully validated three-dimensional biomechanical measurement system. The main strength of the workflow is that it combines hurdle detection with markerless pose estimation, allowing ordinary side-view videos to be converted into hurdle-relative phase labels and technical indicators. This is particularly useful in routine training settings, where low cost, low interference, and rapid feedback are often more important than laboratory-level measurement precision.

It should be noted that camera placement may affect distance-related variables. When the camera is repositioned while still pointing toward the hurdle, deviation from the ideal perpendicular side-view direction introduces projection error. Based on a simplified geometric estimate, camera-angle deviations of 5°, 10°, 15°, and 20° would result in approximate takeoff-distance errors of 0.031, 0.060, 0.087, and 0.112 m, respectively, and landing-distance errors of 0.050, 0.102, 0.155, and 0.211 m. Therefore, the camera should preferably be positioned within ±5° for quantitative distance assessment, whereas deviations within ±10° may be acceptable for general coaching feedback. If the camera orientation remains perpendicular to the running direction and only lateral translation occurs, practical errors are more likely to arise from lens distortion than from projection effects.

However, hurdling is not purely a sagittal-plane movement: Pelvic rotation, mediolateral foot placement, frontal-plane leg motion, and transverse-plane trunk rotation cannot be fully represented from a single side-view camera. This limitation is consistent with previous validation studies showing that single-camera 2D markerless motion analysis is affected by joint visibility and occlusion and that agreement may decrease when relevant anatomical landmarks are not clearly visible [32].

Therefore, the variables extracted in this study should be regarded as practical two-dimensional indicators rather than complete three-dimensional kinematic variables. Future work may extend the current 2D side-view workflow to low-cost 3D markerless analysis using synchronized dual-view smartphone videos. Systems such as OpenCap estimate 3D human kinematics and dynamics from videos captured by two or more smartphones, and Pose2Sim provides a low-cost multi-camera workflow for 3D markerless kinematics [33]. But high-speed and occluded movements such as hurdle clearance still require task-specific validation. A future dual-view framework could therefore help quantify variables not captured reliably in the present side-view method, such as pelvis rotation, mediolateral foot placement, frontal-plane leg motion, and transverse-plane trunk movement.

#### 4.5.2. Future Integration of Temporal Models

The comparison with LSTM and TCN baselines also helps clarify the boundary of the rule-based segmentation strategy. In the present dataset, the rule-based method showed lower overall event-localization error and performed particularly well for takeoff-related events. This suggests that biomechanical rules and hurdle-relative spatial constraints are effective when the athlete performs a recognizable hurdle-clearance pattern. However, for the latter landing-related events, especially in atypical cases, temporal models showed some advantages. This is reasonable because LSTM and TCN models can use sequential context rather than relying only on manually defined event rules. TCNs, in particular, have been reported as strong models for sequence modeling tasks because they can capture temporal dependencies through dilated temporal convolutions [34].

At the same time, the present sample size remains too small to make end-to-end temporal models the primary solution. Deep learning models generally require large and diverse annotated datasets, and data scarcity is a known limitation when training models that must generalize beyond the observed samples [35]. In this context, the rule-based method remains valuable because it is transparent, data-efficient, and directly linked to the biomechanical structure of hurdle clearance. A reasonable future direction is not to replace the current framework with a purely data-driven model but to develop a hybrid strategy. For example, rule-based event constraints could be retained for interpretability, while LSTM, TCN, Transformer, or graph-based temporal models could be used to refine event boundaries, especially during the landing phase and in technically variable athletes. Such a strategy would become more feasible if hundreds or thousands of annotated hurdle-clearance videos were collected across different athletes, hurdle heights, camera conditions, and performance levels.

#### 4.5.3. Practical Value for Field-Based Hurdle Analysis

The broader value of this study lies in its sport-specific integration of visual detection, markerless pose estimation, and biomechanical interpretation. Recent AI-based movement studies have shown that skeletal keypoints, sensor signals, and structured temporal representations can support human activity or lower-limb motion recognition. For example, skeletal-joint image representations and relative-position image methods have been used to encode movement information for recognition tasks [36,37]. Machine learning approaches have also been increasingly applied to posture and movement assessment in broader biomechanical contexts [38]. However, hurdle clearance differs from general activity recognition because the technical meaning of the movement depends strongly on the athlete’s relationship with an external obstacle. The present workflow therefore does not simply classify an action or estimate body keypoints; it uses hurdle position, bar midpoint, and post-reference points to generate hurdle-relative technical variables. This is the main methodological contribution of this study.

Accordingly, the current framework is most suitable for athletes who can already complete a recognizable hurdle-clearance pattern. In such cases, the system can support routine coaching feedback by identifying the takeoff–flight–landing structure and extracting phase-specific indicators that are difficult to quantify by visual observation alone. For athletes with clearly immature or atypical technique, such as a jumping-like clearance pattern or near-simultaneous two-foot landing, biomechanical indicators derived from this framework should be interpreted cautiously. In those cases, direct technical correction by the coach should take priority. Once the athlete’s technique becomes stable enough that major errors are no longer obvious visually, the proposed framework may provide more useful support for identifying subtle technical deficiencies and guiding further refinement.

Overall, the present study should be understood as a step from ordinary video recording toward interpretable, field-based biomechanical feedback. It is not yet a fully validated replacement for three-dimensional motion capture, but it provides a practical and transparent workflow for extracting hurdle-specific information from low-cost monocular videos. Future work should focus on three directions: validating the two-dimensional outputs against three-dimensional reference systems, expanding the annotated dataset to support temporal learning models, and developing hybrid rule-based and data-driven segmentation methods for more robust analysis across different skill levels and movement patterns.

### 4.6. Limitations

This study has several limitations. The proposed workflow was based on monocular two-dimensional side-view video. Therefore, the extracted distance, angle, and center-of-mass-related variables should be interpreted as side-view two-dimensional indicators rather than true three-dimensional kinematic parameters. Although this limitation has been discussed above, it remains an inherent constraint of the present method.

Moreover, the current body17 keypoint scheme does not include detailed foot landmarks, such as the toe and heel. Because touchdown and toe-off events are directly related to foot–ground contact, the present method had to infer these events indirectly from ankle trajectories, knee angle changes, and athlete–hurdle spatial relationships. This may reduce event-detection accuracy when the foot is blurred, occluded, or overlapped with the opposite limb, especially during landing-related events, such as swing-leg touchdown and swing-leg toe-off.

Furthermore, the sample size was relatively limited. The analysis was based on 37 hurdle-clearance videos from student athletes, and the range of competitive levels, movement patterns, and recording conditions was not sufficiently broad to establish full generalizability. Therefore, the current results should be interpreted as method-development and preliminary validation findings rather than definitive evidence of robustness across all athlete populations.

Future studies should expand the dataset across different skill levels, camera conditions, and hurdle heights; incorporate foot-specific keypoints or local foot detection models; and validate the extracted variables against three-dimensional reference systems. With larger annotated datasets, hybrid approaches combining biomechanical rules with temporal learning models may further improve event detection, particularly for landing-phase and atypical movement patterns.

## 5. Conclusions

This study developed an automatic analysis workflow for hurdle clearance based on monocular side-view video, hurdle detection, and markerless human pose estimation. The workflow achieved hurdle position detection, human keypoint extraction, key-event detection, phase segmentation, and phase-specific kinematic variable output. The results showed that the method could divide hurdle clearance into takeoff, flight, and landing phases and extract event-specific indicators such as takeoff distance, flight time, landing distance, center-of-mass rise, joint angles, and trunk angles. Validation results indicated that the system showed good feasibility in human keypoint localization, key-event detection, and extraction of most core variables. By incorporating hurdle detection, human keypoints could be interpreted within a hurdle-relative spatial relationship, thereby extending the application range of simple pose estimation in hurdling technique analysis.

Overall, the proposed method reduces dependence on specialized equipment and manual frame-by-frame processing in hurdling kinematic analysis and may provide a low-cost, low-interference, and repeatable technical analysis solution for field-based training.

## Figures and Tables

**Table 1:** Participant characteristics and hurdle performance.

Variable	Value
Sex	Male: 32; Female: 5
Age (years)	19.51 ± 1.07
Height (cm)	176.76 ± 6.66
Body mass (kg)	69.28 ± 7.39
Hurdling performance (s)	17.91 ± 1.24
Training experience (years)	2.39 ± 1.56

**Table 2:** Frame-wise fused variables and derived descriptors.

Category	Variables	Definition or Role
Person bounding box	bbox_x1, bbox_y1, bbox_x2, bbox_y2, bbox_score	Bounding box and confidence of the detected main athlete.
Body keypoints	bilateral shoulder, hip, knee, ankle coordinates and keypoint scores	Core 2D pose variables used for geometric reconstruction and later kinematic analysis.
Hurdle bounding box	bbox_x1_hurdle, bbox_y1_hurdle, bbox_x2_hurdle, bbox_y2_hurdle, conf	Frame-wise hurdle detection output.
Hurdle reference points	bar_mid_x, bar_mid_y, post_x	External spatial reference used to express athlete–hurdle relative position.
Midpoint variables	pelvis_mid_x, pelvis_mid_y, shoulder_mid_x, shoulder_mid_y	Bilateral midpoint variables derived from left–right hips and shoulders.
Segment-length variables	left_thigh_length_px, left_shank_length_px, left_leg_length_px, right_thigh_length_px, right_shank_length_px, right_leg_length_px, leg_length_px	Frame-wise lower-limb geometric descriptors derived from joint coordinates.
Hurdle geometry variables	box_width_px, box_height_px, box_bottom_y, far_base_top_y, post_base_y, post_height_pixel, hurdle_box_ratio	Derived hurdle geometry variables used for scene scaling and reference construction.
Metric scaling	post_height_real, pixel_to_meter_scale, leg-length variables in meters	Scale conversion variables obtained from hurdle geometry for metric-unit expression.
COM-related variables	com_x, com_y, com_vx_mps, com_vy_mps	Estimated center-of-mass coordinates and velocities derived from fused pose data.
Motion direction	move_dir	Frame-wise movement direction inferred from the fused sequence.

**Table 3:** Rule-based decision logic for the four key events used in event-driven phase segmentation.

Key Event	Biomechanical Role	Decision Logic
Takeoff-leg touchdown	The beginning of the final effective pre-hurdle support.	Pre-hurdle search window Low-position interval of takeoff-leg ankle Local peak of takeoff-leg knee angle Swing-leg knee and ankle ahead of takeoff leg
Takeoff-leg toe-off	The end of takeoff support and the beginning of the flight phase.	After takeoff-leg touchdown Pre-hurdle search window Takeoff-leg ankle low-position interval Takeoff-leg knee-angle peak Takeoff leg behind swing leg Takeoff-leg knee and ankle below hurdle bar
Swing-leg touchdown	The end of the flight phase and the beginning of post-hurdle landing support.	After swing leg crossed the hurdle Vertical acceleration pattern of swing-leg ankle Duration criterion for touchdown segment Earliest valid candidate segment
Swing-leg toe-off	The end of the landing phase and the return to inter-hurdle running.	After swing-leg touchdown Sign change in bilateral ankle vertical difference Pelvis midpoint behind the hurdle Constraint against premature detection
General segmentation standard	Ensures that the segmentation result follows the actual event sequence.	Chronological order: takeoff-leg touchdown → takeoff-leg toe-off → swing-leg touchdown → recovery-leg toe-off Phase labels assigned from adjacent key-event intervals: takeoff, flight, and landing

**Table 4:** Performance of the hurdle detection and segmentation model on the test set.

Class	Images	Instances	Metric Type	Precision	Recall	mAP@0.5	mAP@0.5:0.95
Hurdle	1147	1147	Box	0.969	0.958	0.992	0.942
			Mask	0.955	0.945	0.971	0.755

Note: Images and Instances indicate the numbers of validation images and annotated hurdles, respectively. Box and Mask denote bounding-box detection and segmentation results; mAP@0.5:0.95 is averaged across IoU thresholds from 0.5 to 0.95.

**Table 5:** Pose estimation accuracy by keypoint and key event.

Keypoint Name	T-Leg TD	T-Leg TO	HC	S-Leg TD	S-Leg TO	All
Left shoulder	0.39/100.00	1.32/97.30	1.53/97.30	0.39/100.00	0.40/100.00	0.95/98.92
Right shoulder	0.87/100.00	1.55/94.59	1.04/97.30	0.43/100.00	0.76/100.00	1.00/98.38
Left hip	1.11/100.00	3.32/91.89	1.86/94.59	2.41/94.59	1.20/97.30	2.14/95.68
Right hip	0.43/100.00	2.06/94.59	0.41/100.00	3.38/89.19	1.13/97.30	1.86/96.22
Left knee	0.40/100.00	0.42/100.00	1.05/97.30	0.43/100.00	15.76/94.59	7.07/98.38
Right knee	0.42/100.00	0.41/100.00	3.82/94.59	0.68/100.00	1.49/94.59	1.88/97.84
Left ankle	0.40/100.00	0.42/100.00	0.38/100.00	0.39/100.00	28.40/97.30	12.70/99.46
Right ankle	0.41/100.00	0.44/100.00	38.82/83.78	0.42/100.00	9.45/97.30	17.87/96.22
All	0.61/100.00	1.59/97.30	13.83/95.61	1.52/97.97	11.99/97.30	8.25/97.64

Note: T-leg TD = takeoff-leg touchdown; T-leg TO = takeoff-leg toe-off; HC = hurdle-crossing instant; S-leg TD = swing-leg touchdown; S-leg TO = swing-leg toe-off. Values are presented as RMSE/PCK, where RMSE denotes root mean squared error in pixels and PCK denotes the percentage of correct keypoints under a normalized distance threshold of 0.02. “All” indicates the overall result for the corresponding row or column.

**Table 6:** Frame-wise pose estimation accuracy by keypoint across all video frames.

Keypoint Name	Start (*n* = 322)	Takeoff (*n* = 340)	Flight (*n* = 985)	Landing (*n* = 324)	Continuation (*n* = 269)
Left shoulder	0.39/100	0.56/100	0.41/100	1.21/100	0.41/100
Right shoulder	0.41/100	0.49/100	0.41/100	0.53/100	0.42/100
Left hip	0.4/100	0.4/100	0.82/100	1.16/100	2.56/99.63
Right hip	0.41/100	0.41/100	0.68/100	0.53/100	0.4/100
Left knee	0.42/100	0.45/100	2.94/99.8	10.3/98.46	0.4/100
Right knee	0.4/100	0.42/100	6.31/98.58	6.38/99.38	0.4/100
Left ankle	0.4/100	0.4/100	29.06/91.98	13.9/98.46	0.41/100
Right ankle	0.41/100	0.56/100	2.53/99.8	14.37/99.07	0.4/100

Note: Values are presented as RMSE/PCK. RMSE denotes root mean squared error in pixels, and PCK denotes the percentage of correct keypoints under a normalized distance threshold of 0.02. Start, takeoff, flight, landing, and continuation indicate the temporal intervals used for frame-wise evaluation. The value in parentheses indicates the number of annotated frames included in each interval.

**Table 7:** Comparison of event localization performance between the rule-based method and temporal baseline models (*n* = 37).

Event	Rule-Based	LSTM	TCN
Takeoff-leg touchdown	0.16/0.40/1	3.17/7.26/23	2.94/7.23/23
Takeoff-leg toe-off	0.62/0.85/2	1.81/4.08/13	1.83/4.10/13
Swing-leg touchdown	0.81/1.72/7	1.03/1.38/3	0.84/1.20/3
Swing-leg toe-off	1.49/2.39/12	1.35/1.85/5	1.11/1.40/5
Overall	0.74/1.55/12	1.87/4.31/23	1.70/4.21/23

Note: Values for event localization are reported as MAE/RMSE/Max AE in frames. MAE = mean absolute error; RMSE = root mean square error; Max AE = maximum absolute error.

**Table 8:** Comparison of event localization performance between the rule-based method and temporal baseline models after excluding one special case (*n* = 36).

Event	Rule-Based	LSTM	TCN
Takeoff-leg touchdown	0.19/0.44/1	3.17/7.26/23	2.94/7.23/23
Takeoff-leg toe-off	0.61/0.85/2	1.81/4.08/13	1.83/4.10/13
Swing-leg touchdown	0.64/1.30/6	0.86/1.12/2	0.75/1.07/2
Swing-leg toe-off	1.08/1.61/4	1.17/1.49/3	1.11/1.37/3
Overall	0.63/1.14/6	1.75/4.27/23	1.61/4.25/23

Note: Values for event localization are reported as MAE/RMSE/Max AE in frames. MAE = mean absolute error; RMSE = root mean square error; Max AE = maximum absolute error.

**Table 9:** Summary of core phase-specific kinematic variables.

Phase	Variable	Unit	Mean ± SD	Min	Max
Takeoff	Takeoff distance	m	1.62 ± 0.17	1.35	2.03
	Takeoff support time	s	0.17 ± 0.02	0.12	0.22
	Takeoff-leg knee angle at touchdown	deg	156.96 ± 4.39	147.82	166.84
	Takeoff-leg knee angle at toe-off	deg	160.67 ± 7.06	147.57	174.23
	Trunk angle at takeoff toe-off	deg	13.09 ± 6.61	−0.27	25.4
Flight	Flight time	s	0.44 ± 0.06	0.33	0.55
	COM rise	m	0.19 ± 0.06	0.04	0.31
	Lead knee angle at hurdle crossing	deg	161.72 ± 16.94	130.94	179.69
	Trunk angle at hurdle crossing	deg	30.75 ± 6.74	9.31	43.99
Landing	Landing distance	m	1.18 ± 0.30	0.57	1.81
	Knee angle at landing	deg	164.30 ± 13.77	145.57	179.28
	Shank angle at landing	deg	82.39 ± 7.70	54.01	90
	Trunk angle at landing	deg	14.55 ± 6.33	1.5	30.12
	Recovery time	s	0.12 ± 0.03	0.08	0.18

**Table 10:** Agreement between manually recalculated and system-derived core kinematic variables.

Variable	Manual M ± SD	System M ± SD	MAE	RMSE	ICC(A,1)	Bias (95% CI)	LOA
Takeoff distance (m)	1.62 ± 0.17	1.62 ± 0.17	0.0014	0.0017	1.000	−0.0003 (−0.0008 to 0.0003)	−0.0035 to 0.0030
Takeoff support time (s)	0.17 ± 0.02	0.17 ± 0.02	0.0123	0.0164	0.738	0.0083 (0.0035 to 0.0132)	−0.0198 to 0.0365
Takeoff-leg knee angle at touchdown (deg)	156.77 ± 4.35	156.96 ± 4.39	0.6059	0.7529	0.985	0.1945 (−0.0537 to 0.4426)	−1.2526 to 1.6415
Takeoff-leg knee angle at toe-off (deg)	161.04 ± 7.10	160.67 ± 7.06	0.7882	1.6543	0.973	−0.3704 (−0.9205 to 0.1797)	−3.5780 to 2.8371
Trunk angle at takeoff toe-off (deg)	12.86 ± 6.36	13.09 ± 6.61	0.4604	0.9075	0.990	0.2267 (−0.0603 to 0.5138)	−1.5193 to 1.9727
Flight time (s)	0.44 ± 0.06	0.44 ± 0.06	0.0176	0.0304	0.864	−0.0023 (−0.0122 to 0.0077)	−0.0626 to 0.0581
COM rise (m)	0.21 ± 0.06	0.19 ± 0.06	0.0228	0.032	0.865	−0.0209 (−0.0289 to −0.0128)	−0.0692 to 0.0274
Lead knee angle at hurdle crossing (deg)	165.94 ± 11.06	166.33 ± 10.85	0.8085	1.3509	0.992	0.3825 (−0.0664 to 0.8315)	−2.1963 to 2.9614
Trunk angle at hurdle crossing (deg)	31.92 ± 6.54	30.75 ± 6.74	2.1094	3.3742	0.872	−1.1752 (−2.2084 to −0.1419)	−7.4601 to 5.1098
Landing distance (m)	1.18 ± 0.30	1.18 ± 0.30	0.0013	0.0016	1.000	0.0004 (−0.0001 to 0.0009)	−0.0026 to 0.0034
Knee angle at landing (deg)	166.05 ± 8.42	166.14 ± 8.13	0.6797	1.1863	0.990	0.0942 (−0.2975 to 0.4860)	−2.2565 to 2.4450
Shank angle at landing (deg)	82.39 ± 7.77	82.39 ± 7.70	0.3050	0.5165	0.998	0.0042 (−0.1645 to 0.1730)	−1.0221 to 1.0306
Trunk angle at landing (deg)	14.40 ± 6.71	14.55 ± 6.33	0.7101	1.2862	0.981	0.1473 (−0.2701 to 0.5647)	−2.3916 to 2.6863
Recovery time (s)	0.13 ± 0.02	0.12 ± 0.03	0.0223	0.0270	0.211	−0.0061 (−0.0152 to 0.0030)	−0.0583 to 0.0462

Note: ICC(A,1) = two-way mixed-effects, absolute-agreement, single-measure intraclass correlation coefficient. Bias is presented with 95% confidence intervals. LOA = limits of agreement.

## Data Availability

The data presented in this study are not publicly available due to privacy restrictions related to identifiable human movement videos. Processed data and analysis code are available from the corresponding author upon reasonable request.

## Abbreviations

The following abbreviations are used in this manuscript:

CNN	Convolutional neural network
COM	Center of mass
DLT	Direct linear transformation
HC	Hurdle-crossing instant
LSTM	Long short-term memory
MAE	Mean absolute error
mAP	Mean average precision
ICC	Intraclass correlation coefficients
LOA	Limits of agreement
CI	Confidence interval
PCK	Percentage of correct keypoints
RMSE	Root mean square error
RTMDet	Real-time multi-person detector
RTMPose	Real-time multi-person pose estimation model
S-leg TD	Swing-leg touchdown
S-leg TO	Swing-leg toe-off
T-leg TD	Takeoff-leg touchdown
T-leg TO	Takeoff-leg toe-off
